# The Localized Ionic Microenvironment in Bone Modelling/Remodelling: A Potential Guide for the Design of Biomaterials for Bone Tissue Engineering

**DOI:** 10.3390/jfb14020056

**Published:** 2023-01-19

**Authors:** Yuqing Mu, Zhibin Du, Lan Xiao, Wendong Gao, Ross Crawford, Yin Xiao

**Affiliations:** 1 Centre for Biomedical Technologies, School of Mechanical, Medical and Process Engineering, Queensland University of Technology (QUT), Brisbane, QLD 4000, Australia; 2 The Australia-China Centre for Tissue Engineering and Regenerative Medicine (ACCTERM), Queensland University of Technology (QUT), Brisbane, QLD 4000, Australia; 3 School of Medicine and Dentistry & Menzies Health Institute Queensland, Griffith University (GU), Gold Coast, QLD 4222, Australia

**Keywords:** intrinsic osteoinductivity, inorganic biomaterials, localized ionic microenvironment, passive osteoinductivity

## Abstract

Bone is capable of adjusting size, shape, and quality to maintain its strength, toughness, and stiffness and to meet different needs of the body through continuous remodeling. The balance of bone homeostasis is orchestrated by interactions among different types of cells (mainly osteoblasts and osteoclasts), extracellular matrix, the surrounding biological milieus, and waste products from cell metabolisms. Inorganic ions liberated into the localized microenvironment during bone matrix degradation not only form apatite crystals as components or enter blood circulation to meet other bodily needs but also alter cellular activities as molecular modulators. The osteoinductive potential of inorganic motifs of bone has been gradually understood since the last century. Still, few have considered the naturally generated ionic microenvironment’s biological roles in bone remodeling. It is believed that a better understanding of the naturally balanced ionic microenvironment during bone remodeling can facilitate future biomaterial design for bone tissue engineering in terms of the modulatory roles of the ionic environment in the regenerative process.

## 1. Introduction

A localized microenvironment in bone remodeling milieus is generated and maintained when ions and biological molecules are released during the demineralization and degradation of bone matrix by protons and proteases secreted by osteoclasts, respectively, and bone formation by osteoblasts [1]. However, the localized microenvironment will be altered at implantation sites, with biomaterials interacting with extracellular fluid and cells. Considered vehicles for localized delivery of inorganic ions and ionic groups, inorganic biomaterials are no longer merely an inert scaffold but a reservoir for bioactive cues for modulating the bone remodeling process [2,3,4].

Inspired by the abundance of elements in the biological system and the effects of nutritional deficiency or overload, therapeutic applications of bioinorganic ions have been explored for many years. For example, the platinum drug cisplatin has been used for cancer treatment, the gold drugs myocrisin and auranofin for rheumatoid arthritis treatment, silver compounds in the pharmaceutical industry for their antimicrobial properties, and lanthanides and some transition metals as radiopharmaceuticals and diagnostic agents [5,6,7]. Meanwhile, the non-scientific and unregulated usage of inorganics can sometimes also be poisonous and lead to tragic disorders or diseases. For example, grey-colored skin is caused by unsafe nasal sprays due to the precipitation of silver salts, and copper deficiency results from over-supplemented zinc for prostate problems and acne [4,5]. In the field of regenerative medicine, the roles of elements in modulating cellular activities have gradually been unraveled, either as essential cofactors of enzymes and proteins or as regulatory molecules in ion channels or secondary signaling. Uncovered biological roles of ions provided possibilities to explore the applications of inorganic biomaterials in hard and soft tissue engineering by acting as vehicles to deliver ions and ionic groups locally.

Among all, calcium phosphates (CaPs) based on inorganic biomaterials are one of the most extensively studied types for bone grafting. They are composed of calcium ions and phosphate groups, which are omnipresent in the bloodstream or fixed in the bone mineral phase [8,9,10]. These synthetic bone substitutes can bind with natural bones by forming a solid biomaterial-bone interface, lacking osteoinductive and angiogenic properties [3,11,12,13,14]. Significant progress has been made in designing functional CaPs-based biomaterials with: (a) optimized geometry, roughness, and appropriate porosity for entrapping and concentrating growth factors or osteoprogenitor cells via proteins that could enhance cell adhesion, (b) incorporated growth factors or proteins that could modulate cellular activity, (c) doped trace elements that enhance osteogenesis in vitro [15,16]. However, the clinical performances of current CaP-based biomaterials are still unsatisfactory and incomparable to autologous bone grafts due to low bioactivities [8,10]. Nevertheless, the optimization of CaPs-based biomaterials significantly boosted the understanding of the modulatory effects of ions in the biological system [3,4]. Considering the abundance of ions in the bone environment and the current knowledge of their modulatory roles in maintaining the bone remodeling balance, it is expected that a deeper understanding of ions in the bone environment would provide new insights to guide the future design of inorganic biomaterials for bone tissue engineering [3,17]. In this review, we focus on inorganic components in the bone environment, helping to provide new insights on how it might be profound to guide the future design of inorganic biomaterials for bone tissue engineering.

## 2. Bone Mineral Phase and Localized Ionic Microenvironment

**Figure 1:**
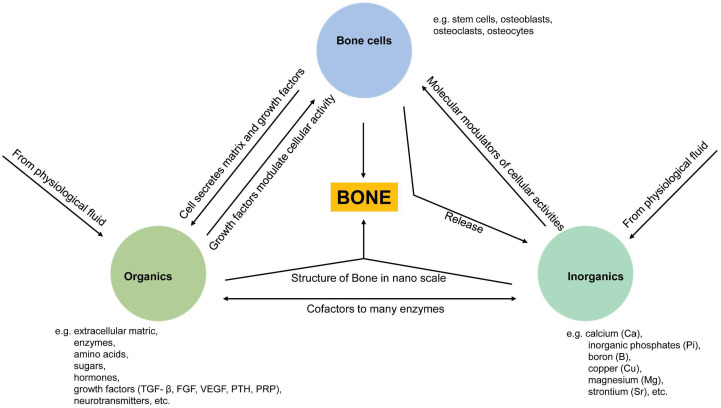
Schematic illustration of relationships between three essential components (cells, organics, inorganics) in maintaining bone homeostasis. Cell-derived organic molecules, such as growth factors and enzymes, modulate cellular activities; Osteoclasts release ions from the bone matrix during bone resorption, and in turn, ions act as molecular modulators of cellular activities and as components of apatite crystals being deposited into the bone matrix with the modulation of cells; Ions are co-factors to many enzymes, and ions are immobilized as apatite crystals into collagen fibrils from the bone structure at the nanoscale.

**Figure 2:**
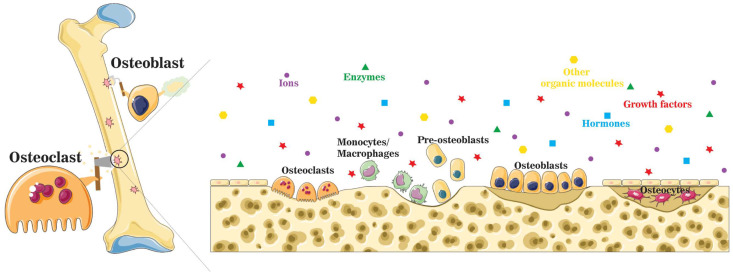
A schematic illustration of the localized microenvironment at the bone remodeling site. Bone homeostasis is maintained by the balance between bone formation by osteoblasts and bone resorption by osteoclasts. During the bone remodeling process, organic molecules, such as enzymes, growth factors, and hormones, are released into the localized microenvironment, together with a mixture of inorganic components.

Bone homeostasis is maintained in a series of highly complicated events orchestrated by: (a) interactions among different types of cells, mainly mesenchymal stem cells (MSCs), osteoprogenitor cells, osteoblasts, osteoclasts, and osteocytes, and (b) interactions of cells with extracellular matrix in a localized microenvironment, and (c) interactions of cells with components in surrounding biological milieus, such as organics (amino acids, enzymes, hormones, fatty acids, neurotransmitters, sugars, vitamins, etc.), inorganics (inorganic ions or groups, such as calcium, phosphate, potassium, sodium, carbonate, etc.), as well as waste products from cell metabolism (Figure 1) [9]. Naturally, the localized ionic microenvironment is maintained by the balance between bone-forming cells, osteoblasts, and bone-resorbing cells, osteoclasts, during the bone remodeling process [18] (Figure 2). Specifically, osteoclasts firmly attaching to the bone surface could achieve a pH fall to a limit value of pH 3.0 or less for dissolving the bone mineral and favor collagen degradation by secreting lysosomal proteinases [19]. Organics in this microenvironment have been extensively studied since the last century, especially cell-secreted growth factors that play roles in bone formation, such as TGF-β (transforming growth factor-beta), FGF (fibroblast growth factor), BMP (bone morphogenetic proteins), IGF-I (insulin-like growth factors I), etc. [20]. Inorganics in this microenvironment have also been extensively studied because they are essential for the bone mineral formation, and quality of the mineralized tissue, either liberated from bone or circulating in body fluid. Moreover, ions from the localized microenvironment are now considered to consist of crystal components and molecular modulators in many biological processes in bone remodeling, i.e., bone formation and resorption [3,9,21,22]. The list of inorganic ions and ionic groups that affect bone metabolism and homeostasis as signaling molecules has dramatically increased in the past decades. More previously less-studied elements in the periodic table have been surprisingly found to play a role in the etiology and pathogenesis of some bone diseases or the modulation of cellular activities, especially metallic elements, because they are prone to lose electrons to form positively charged ions and tend to dissolve in biological fluids or be attracted by negatively charged biological molecules, proteins, or DNAs, to form active metal complexes [3,4,23].

Bone mineral, known as biological apatite, is incorporated in collagen fibrils, arranged with a *c*-axis parallel to the direction of fibrils, with lengths of 30–50 nm, widths of 15–30 nm, and thicknesses of 2–10 nm [24]. Biological apatite has been modeled as hexagonal carbonated hydroxyapatite based on X-ray diffraction (XRD) results, with the lattice parameters of a = b = 9.432 Å, c = 6.881 Å, and γ = 120°. Hydroxyl ions (OH^−^), parallel to the *c*-axis, are positioned on the screw axes at every one-half of the unit cell, pointing in opposite directions to neighboring OH^−^s. Tetrahedral phosphate ions (PO_4_^3−^), immobilized by calcium ions (Ca^2+^) interspersed among them, as well as marginal calcium ions (Ca^2+^), shared with neighbor unit cell. Notably, steric interference between adjacent OH^−^s in hexagonal hydroxyapatite unit cells could be overcome by vacancy or replacement of an OH^−^ by impurity ions, the most likely event in organisms, or by conversion of hexagonal to monoclinic space group at high temperature, rearranging adjacent OH^−^s to a uniform direction [25,26].

**Figure 3:**
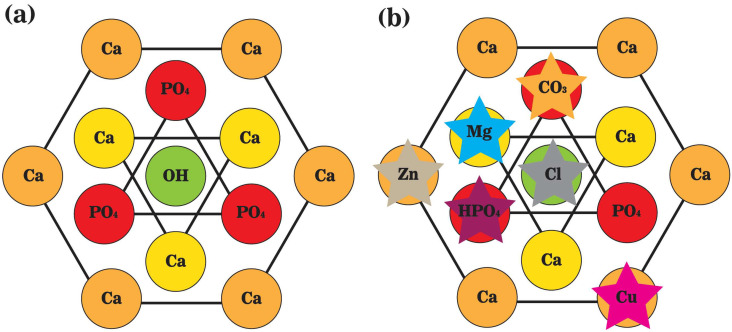
A schematic representation of the top view of unit cells of (**a**) stoichiometric hydroxyapatite; and (**b**) biological apatite crystals. Hydroxyl ions (OH^−^) are positioned on the screw axes at every one-half of the unit cell, paralleling the *c*-axis. Calcium ions (Ca^2+^) are interspersed among tetrahedral phosphate ions (PO_4_^3−^), and the marginal ones are shared with neighbor unit cells.

The accumulation of most inorganics in the body can be attributed to the formation of apatite crystals in bone, with distinguished content and composition among species and individuals, resulting from differed preferences on elements in different species, variations in diet, and relative abundance in the environment [27,28]. During bone resorption, ions and ionic groups will be liberated from CaP based network into the local microenvironment in acidic conditions, participating in local bone remodeling or being carried away by physiological fluid. Therefore, ions and ionic groups entering the localized microenvironment are determined by the composition of bone minerals and vice versa. Specifically, the content and level of ions and ionic groups in the localized biological milieu affect the formation of the bone mineral through ionic substitution and, consequently, the properties of the final crystalline product in the mineral phase [29,30,31,32]. For example, OH^−^ (minor site) or PO_4_^3−^ (primary site) sites could be replaced by CO_3_^2−^, forming type A and B carbonated hydroxyapatite, respectively. PO_4_^3−^ site could also be replaced by hydrolyzed phosphate (HPO_4_^2−^) structure in mature bone, while OH^−^ could be substituted with florin (F^−^), chlorin (Cl^−^) ion, or orthosilicic acid (SiO_4_^4−^) structure [29,30,32]. Moreover, ionic substitutions also happen where Ca^2+^ is replaced by other metals, such as sodium (Na^+^), potassium (K^+^), magnesium (Mg^2+^), zinc (Zn^2+^), manganese (Mn^2+^), cobalt (Co^2+^), strontium (Sr^2+^), iron (Fe^2+^), copper (Cu^2+^). Ionic exchange in biological apatite alters crystalline structures, resulting in modified crystal size, growth rate, and properties. Compared with stoichiometric or geological apatite crystals, biological apatite crystals have smaller crystallite sizes, less ordered crystal structure, lower crystallinity, and higher solubility (Figure 3) [3,33]. The influences of different ions or ionic groups on biological apatite crystals are balanced by each other. For example, substitutions of PO_4_^3−^ by CO_3_^2−^ and of Ca^2+^ by Zn^2+^ or Mg^2+^ inhibit crystal growth, increase crystal disorder and solubility, and lower the crystallinity [29,30,34]. Replacements of Ca^2+^ by Al^3+^, La^2+,^ or Fe^2+^ accelerate crystal growth, and replacement of OH^−^ by F^−^ on the lattice reduces the solubility [29,30,34]. Additionally, replacing OH^−^ with SiO_4_^4−^ causes a contraction on the *a*-axis and an expansion on the *c*-axis of the crystal lattice [35]; replacing Ca^2+^ with Sr^2+^ causes an expansion on both the *a-* and *c-axes* [4].

**Figure 4:**
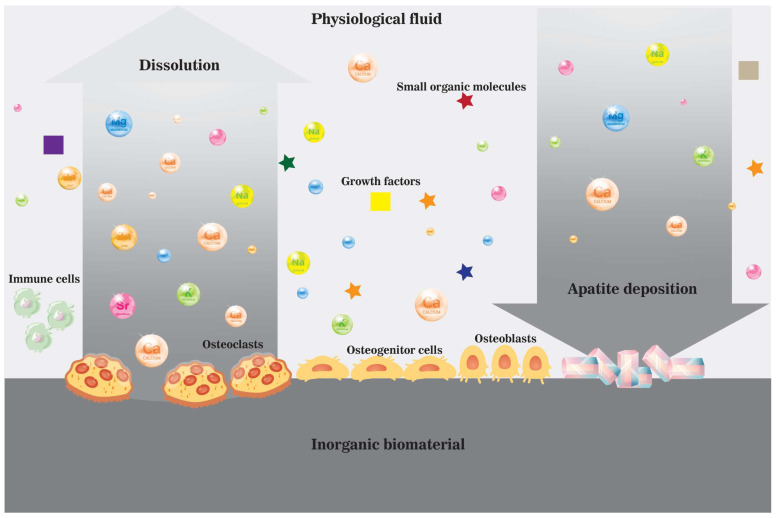
A schematic illustration of the dissolution and precipitation process near the surface of an inorganic biomaterial in vivo in the ionic microenvironment created by cells and physiological fluid enriched by dissolved biomaterial.

During bone trauma, such as a fracture, bone healing starts with the invasion of blood into the traumatic space. A microenvironment is formed along with blood clots and calluses, where cells interact with components in the extracellular matrix and extracellular fluid. However, the localized microenvironment will be altered at the implantation site with the involvement of inorganic biomaterials due to extensive interactions between biomaterials and the microenvironment [16]. The contribution of inorganic biomaterial at the implantation site to the healing process can never be underestimated because many biomaterial intrinsic features, including parameters (composition, structure, topography), and properties (crystallinity, dissolution profile, surface charge), can make a difference in the localized microenvironment and cellular interactions, as well as cellular activities, and consequently the bone formation process (Figure 4) [16]. Therefore, understanding the influence of biomaterials on components in the localized ionic microenvironment shall guide the design of future inorganic biomaterials for bone grafting. Active roles of ions as molecular modulators upon many cellular activities during bone remodeling provide the material with more possibilities other than structural support and protein/cell entrapping.

## 3. Active Osteoinductivity of Inorganic Biomaterials and Enriched Localized Microenvironment

There are a variety of commercial substitute materials for bone and tooth repair/replacement, including metals, polymers, corals, processed human or animal bones, synthetic CaP materials such as ceramics or cement, and hybrid composites [16]. CaPs are one of the most extensively studied inorganic materials for bone grafting due to the omnipresent presence of calcium ions and phosphate groups in the bloodstream or bone. They are excellent in biocompatibility, osteoconductivity, and osteointegration but are brittle and unsuitable for load-bearing [36]. The first attempt to repair surgically created defects in rabbits with artificial CaP material (TCP) was in 1920 [37]. In 1975, β-TCP was applied for the first time in a surgically created periodontal defect in dogs and as an adjunct to apical closure in pulpless permanent teeth in humans [38,39,40]. The first attempt to replace tooth roots with synthetic dense HAp cylinders was reported in 1979 [41]. However, the popularity of CaPs as substitute xenografts or allografts did not start until the late 1990s, which were strictly controlled due to the consequent appearance of diseases after implantation, such as acquired immunodeficiency syndrome (AIDS), and bovine spongiform encephalopathy [2,23,42,43].

Bioactive glasses with a modified SiO_2_ network, developed by Larry L. Hench in the late 1960s, are another group of extensively investigated inorganic biomaterial as an implant for bone defects over the years and achieved great success in the clinical field [44]. Many commercially available synthetic inorganic biomaterials are primarily obtained via wet chemistry, starting from a mixture of ionic solutions or solid-state conversion with heat treatment. In addition to the synthetic method via wet chemistry, inorganic biomaterials can also be obtained from nature. For example, CaP-based biomaterials could be obtained from chemically similar marine coral via hydrothermal conversion of the calcium carbonate skeleton of marine coral to hydroxyapatite. Coral-derived hydroxyapatite has been used as a bone graft since the 1980s for good biocompatibility and structural support. Still, it was limited in clinical practice due to inherent weak mechanical strength and low degradability [45,46]. Moreover, the composition of marine coral-derived hydroxyapatite also differs from that in the natural bone mineral phase [47]. Elements in the bone mineral phase are constantly fixed and liberated during bone remodeling. Ions involved in amorphous calcium phosphate formation at the early stage of nucleation, phase transition during crystallization, and extensive ionic substitutions along the mineralization process during bone formation, are from the local microenvironment at the implantation site, i.e., bloodstream and implanted material. These ions are essential to the body and are considered bioactive ions not only because they form the bone mineral phase through crystallization, but also because they participate in modulating multiple cellular activities in bone metabolism, such as the proliferation and differentiation of osteoblasts and osteoclasts, as well as the responses of immune cells [48,49,50,51,52,53,54]. Therefore, it is speculated that biomaterials obtained from bone are most likely to achieve the maximum retainment of bioactive trace elements by retaining bioactive ions originally present in bone. There are several bone mineral products on the market, mainly in dentistry, such as Cerabone (AAP Biomaterials GmbH, Berlin, Germany) and Bio-Oss (Geistlich Pharma AG, Wolhusen, Switzerland) [11]. Cerabone^®^, a bone mineral product of bovine origin manufactured by a proprietary 1200 °C production process, is mainly used to support the successful placement of dental implants. And Bio-Oss^®^, primarily used in dental surgery, is obtained by removing organic substances with a stepwise annealing process up to 300 °C, followed by a strong alkali treatment [43,55]. Removal of viruses, bacteria, proteins, and other organic substances via sintering not only leaves a three-dimensional porous network, facilitating protein adsorption and cell adhesion but also increases the crystallinity of mineral crystals with reduced solubility and improved mechanical strength and biological stability. However, the degradation of this product type is considerably slow, with visible remnants in the 30-month post-implantation [56].

After implantation, graft materials are expected to allow bone-like apatite to deposit on the surface-mediated by cells, and consequently bond to surrounding living bone, obtaining extra stabilization and fixation at the implant region [57,58]. Currently, the most widely adopted approach to predict bone-bonding ability (i.e., osteoconductivity) is to test the ability to deposit bone-like apatite on the surface of a material by immersing in simulated body fluid (SBF), an ionic solution with nearly equal ion concentrations to those of human blood plasma [57]. Graft materials are also expected to be excellent in osteointegration, as biodegradation and biosorption favor vascular and bony ingrowth and cellular waste removal. In addition to osteoconductivity and osteointegration, osteoinductivity, the ability to induce new bone growth, is another essential property of graft material. The osteoinductive property of a biomaterial is usually demonstrated by de novo bone formation in the absence of osteogenic factors and non-osseous sites after implantation in vivo.

In general, osteoinductivity of inorganic biomaterials can be obtained from (a) material design with proper geometry, roughness, and porosity that facilitates bone growth by entrapping and concentrating growth factors or osteoprogenitor cells, (b) incorporation with growth factors, bioactive proteins or trace elements that would induce bone growth [3,59]. If osteoinductivity obtained through the optimization of parameters and biophysical properties of an inorganic biomaterial via entrapped or concentrated growth factors or osteoprogenitor cells is considered “passive osteoinductivity”, osteoinductivity obtained by incorporating osteogenic proteins or bioactive inorganics should be considered as “intrinsic osteoinductivity”, because molecules, such as growth factors, and bioactive trace elements, liberated from materials with active osteoinductivity participate in new bone formation proactively via modulating cellular activities.

Osteoinductivity of demineralized bone matrix in different animals was reported in 1965, and osteogenic factors originally present in the matrix, specifically bone morphogenetic proteins (BMPs), were demonstrated later [60,61]. Inorganic biomaterials incorporated with BMPs, sourced from extraction or recombinant procedures, have been investigated extensively for many years due to their excellent osteoinductivity [62,63,64,65,66]. Other biological osteogenic/angiogenic factors have also been extensively studied over the years, such as TGF-β, FGF, VEGF (vascular endothelial growth factors), parathyroid hormone, and PRP (platelet-rich plasma) [3,15,23,67,68,69]. Numerous combinations of growth factors and types of inorganic biomaterials have been explored for intrinsic osteoinductivity and angiogenesis, as summarized in many reviews [10,16,68,70]. However, inorganic biomaterials incorporated with growth factors are mainly limited in clinical application, with increasingly raised safety concerns regarding the off-label usages of growth factors and their high costs [4].

With intrinsic properties of inorganic biomaterials to release ions, osteoinductive inorganic biomaterials can also be achieved via increased ion concentration in the localized microenvironment. In general, the liberation of ions from biomaterials is believed to enrich the ionic microenvironment, alter ion concentrations and local pH and get involved in bone formation by increasing the supersaturation of ions toward the deposition of hydroxyapatite or as molecular modulators to affect cell signaling and activities [3,4,16,54]. The mineralization on the surface of inorganic biomaterial after implantation, as well as the process of bone formation, is affected by cytotoxicity and osteoconductivity of the material and the impact of it upon cellular activities by releasing ions and ionic groups into the local microenvironment, i.e., the biological milieus. A schematic illustration is shown in Figure 4 to explain the dissolution and precipitation process near the surface of biomaterial in vivo in the ionic microenvironment created by physiological fluid and enriched by dissolved biomaterial. Specifically, ions and ionic groups are liberated from biomaterial either through solubility-determined dissolution in the physiological environment or cell-mediated dissolution in the acidic environment created by macrophages or osteoclasts, resulting in localized supersaturation of inorganics in the microenvironment, further leading to the precipitation of calcium-deficient HAp [4,8,16]. Inspired by the observed integration of biomaterials with the host bony tissues via the deposition of HAp, simulated body fluid (SBF) was developed to predict in vivo bone-bonding activity near the surface of the implanted biomaterial [8,57]. The standardized SBF solution contains a similar ionic profile as the blood and showed a good correlation between the in vivo bioactivity of bioactive glass and apatite-forming ability in the early years [57,71]. Some concerns were proposed in recent years regarding the validity of the SBF immersion test by Bohner et al. and Pan et al. [72,73]. For example, the interference of proteins on apatite formation and the control of carbonate content is not considered [72]. In addition, the roles of ions and ionic groups in the localized biological milieu are also underestimated because they are never merely components in forming mineral crystals, aggregating freely to reach a relatively stable state with lower energy, but also modulators of various cellular activities, such as the proliferation and differentiation of osteoblasts/osteoclasts and getting involved in the crystal formation process on the surface of biomaterials [4,8,16].

## 4. Summary of Ions and Ionic Groups in the Maintenance of Bone Homeostasis

**Figure 5:**
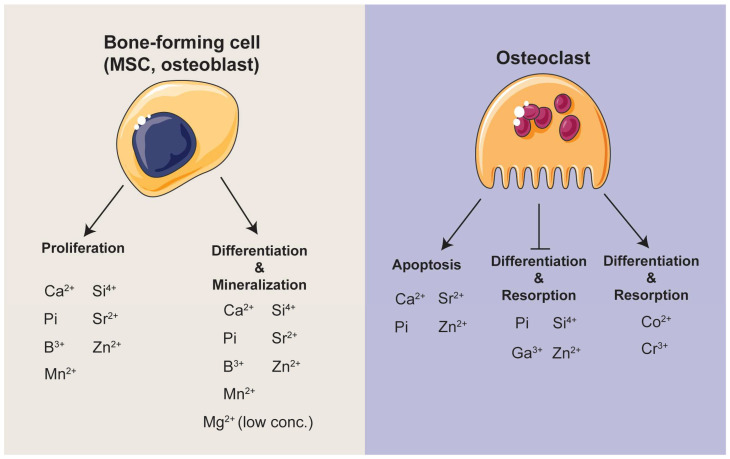
An illustration of the current understanding of the influence of ions on MSCs, osteoblasts, and osteoclasts.

Inorganic ions are not only nutrients in the body but also have the potential as components in diagnostic or therapeutic agents to study or treat various diseases and metabolic disorders, explaining why they have great potential to affect bone regeneration to a similar extent as recombinant growth factors but free from safety issues [4,23]. For example, calcium and phosphate are essential in bone and many other biological processes. Sufficient calcium intake from food or supplementation contributes to maintaining calcium homeostasis in the body, promotes mineralization during growth, and reduces bone loss in the elderly. In contrast, prolonged calcium deficiency may lead to rickets, osteomalacia, and osteoporosis [74]. Similarly, long-term inorganic phosphorus deficiency causes hypophosphatemia, impaired bone mineralization, dysfunction in the blood, muscle, and central nervous system, and the cardio and respiratory system [75]. In skeletal bone, the local availability of both ions is one of the determinants for extracellular matrix mineralization rate, the last step of the bone formation process, and regulatory molecules for multiple cellular activities. However, the bioactivities of other ions were significant and cannot be overlooked. The biological influences of ions at both physiological and cellular levels have been summarized in Table 1 and Figure 5. Notably, there are some limitations in these studies and should be considered in future studies: (a) the ionic profile in the cell culture media upon the addition of ions was overlooked in most studies; (b) the discrepancy/consistency between in vitro and in vivo studies should be emphasized; (c) the justification of whether phenomenon observed in the investigation is caused by the ion of interest.

### 4.1. Extracellular Calcium-Ca^2+^

Extracellular Ca^2+^ has been shown to correlate with multiple cellular activities of MSCs (growth, osteogenic differentiation, and mineralization), osteoblasts (survival, proliferation, and differentiation), and osteoclasts (survival and bone resorption activity) via a variety of intracellular signaling pathways in vitro [4,54,164,165]. In MSCs, the optimized concentration of Ca^2+^ is 1.8 mM, the same concentration supplied in culture media to maintain cell growth [76]. The microenvironment of Ca^2+^ at a concentration <1.8 mM significantly impeded cell growth and osteogenic differentiation [76]. Higher Ca^2+^ concentration, on the other hand, showed no additional promotive effect on cell growth but affected the extent of cell mineralization in a dose-dependent manner [76]. The fluctuation of extracellular Ca^2+^ concentration is most likely to be sensed by the functional calcium-sensing receptor (CaSR) on the cell membrane, which is a member of the G-protein-coupled receptor (GPCR) superfamily [79,164,165]. CaSR is believed to be critical in maintaining the homeostasis of extracellular Ca^2+^ concentration and modulating cell metabolism in many cells, such as parathyroid gland cells, kidney cells, bone cells, endothelial cells, and stem cells [164,166]. In osteoblast cells, Ca^2+^ has been suggested to promote osteoblast proliferation and survival (2–4 mM), as well as differentiation (~5 mM), with elevated expression of several osteogenic markers such as type I collagen (Col-I), bone morphogenetic proteins (BMP), osteocalcin (OCN), etc., most likely via CaSR-mediated signaling pathways [77,78,164,165,167,168,169,170,171]. The proliferation of osteoblast is associated with the activation of extracellular signal-regulated kinase-1 and -2 (ERK-1 and ERK-2) signaling pathways from the mitogen-activated protein kinase (MAPK) superfamily through dual phosphorylation of critical threonine and tyrosine residues [77,78]. The inhibition of osteoblast apoptosis is attributed to the activation of the phosphatidylinositol 3-kinase (PI3K)/protein kinase B (PKB) pro-survival pathway [77,78]. Other intracellular signaling pathways, such as phospholipase C (PLC) and protein kinase C (PKC), Jun-terminal kinase (JNK), and cyclic adenosine monophosphate (cAMP)/protein kinase A (PKA), are also activated through CaSR in high Ca^2+^ environment to affect cell survival [69,172,173]. In addition, the expression of several secondary messengers can also be induced by CaSR signaling pathways, mediating extracellular Ca^2+^ level and controlling osteoblast cell fate, such as insulin-like growth factor (IGF)-II (required for the subsequent cell proliferation), or prostaglandin E_2_ (PGE_2_) produced by cyclooxygenase-2 (COX-2) (associated with alkaline phosphatase activity, and the expression of osteocalcin) [173,174,175]. Moreover, Ca^2+^ has also been shown to regulate cell morphology via cell-cell or cell-matrix interaction, enhancing the expression of angiopoietin-1 (Ang1) and angiogenesis [166,167,176]. In osteoclasts, internalized through CaSR, Ca^2+^ has been shown to sequentially activate the PLC signaling pathway, followed by PLC-dependent translocation of nuclear factor-κB (NF-κB) from the cytoplasm to the nucleus of mature osteoclasts and consequently induce cell apoptosis to inhibit bone resorption [9,79,80].

### 4.2. Inorganic Orthophosphate—Pi

The homeostasis of phosphate in the body is maintained by the cooperation of the gut, bones, and kidneys and balanced by parathyroid hormones, but limited knowledge from the entry beyond regarding the sensing mechanism and consequent proposal for appropriate regulation cascade [75,177]. The optimized concentration of Pi for MSC growth in vitro was proposed to be 0.09 mM; either higher or lower concentration caused impeded growth but showed little effect on cell differentiation or mineralization [76]. In cultured osteoblasts, Pi is found to be involved in modulating cell proliferation and DNA synthesis in a dose-dependent manner at a concentration from 2 to 4 mM, in part via the IGF-1 signaling pathway [178,179]. Pi is also found to regulate osteoblast differentiation and bone mineralization. For example, it induces the production of osteopontin (OPN), a molecule involved in the regulation of bone mineralization, through the activation of both ERK1/2- and PKC- dependent signaling pathways, as well as alkaline phosphatase (ALP) activity in vitro [180]. Pi stimulates the expression of stanniocalcin 1 (STC1, a regulator for the accumulation of transcription factor), pituitary-specific positive transcription factor 1 (Pit1), and consequently increases Pi uptake and mineralization both in vitro and in vivo [82]. In addition, it stimulates the production of matrix Gla protein (MGP, one of the key regulators in extracellular mineralization) with the involvement of Ca^2+^ via ERK1/2- dependent signaling pathways and upregulates the expression of Fos-related antigens 1 and 2 (Fra-1/2) of activator protein-1 (AP-1) family in vitro [181,182,183]. The Pi-promoted osteoblastic differentiation and mineralization provided theoretical support for the localized delivery of Pi from implant materials to promote mineralization [81,82]. However, a high Pi microenvironment resulting from bone resorption or material dissolution may cause significant osteoblast apoptosis through the induction of a transition on mitochondrial membrane permeability, in accordance with observed osteoblast cell apoptosis at bone resorption sites [50,83]. In osteoclasts, osteoclastic bone resorption is well known to be stimulated by low Pi concentration but inhibited with the increase of Pi level [84,184]. The inhibitory effect of Pi on the bone resorptive activity at higher concentrations can be partially attributed to the direct induction of osteoclast cell apoptosis and the inhibition of receptor activator of nuclear factor kappa-Β ligand (RANKL)-induced JNK and Akt signaling pathways [84,85].

### 4.3. Other Bioactive Inorganic Ions

Boron (B), an essential micronutrient, is considered to play an important role in the maintenance of bone and osteogenesis. Several in vivo studies have shown that B ion deficiency could result in reduced osteogenesis, and B ion deprivation would inhibit bone formation, resulting in reduced bone volume and mechanical strength. Beneficial effects on bone micro-architecture and strength could be observed with the nutritional intake of B [87,88,89,90]. In addition to the effects of dietary B from previous in vivo studies, beneficial effects of B ion are also found at the cellular level in BMSCs and osteoblasts: B ion is capable of increasing osteogenic marker gene (*ALP*, *OCN*, and *Col-I*) expression and inducing early matrix mineralization in MSCs, and regulating osteogenic marker expression (runt-related transcription factor 2 (Runx2), and bone sialoprotein (BSP) at mRNA level, BMP-4, -6 and -7 at protein level) in osteoblasts [49,91,92].

Copper (Cu) is an essential trace element required for the function of several important enzymes in the body, and it is necessary to maintain bone quality and strength [185]. Cu^2+^ deficiency causes abnormal bone formation with impaired quality as a co-factor of an enzyme, lysyl oxidase [93]. It prevents crosslinking between structural proteins, collagen, and elastin; At the same time, excess Cu levels may generate free radicals, inducing lipid peroxidation and affecting bone metabolism, and may lead to severe neurological issues or liver diseases [93,94,95]. Nevertheless, being discovered as an essential element with angiogenic and innate antibacterial properties, the applications of Cu^2+^ have attracted much attention in biomaterial fabrication [4,96,97,98]. Rapid and enhanced vascularization and increased extracellular matrix formation are achieved with several Cu-doped biomaterials, bringing novel insights to the traditional concept of accelerating bone formation by filling pores instead of ingrowth from periphery regions [99].

Gallium (Ga) is not an essential element in the body but positively affects bone formation with profound anti-resorptive activity [100]. Ga ion is found to inhibit osteoclast differentiation and osteoclastic resorptive activity in a dose-dependent manner by blocking the transient receptor potential cation channel subfamily V member 5 (TRPV5) Ca^2+^ channel (essential for osteoclast bone resorption); Improved mineralization and elevated mechanical properties results from inhibited expression of nuclear factor of activated T cells, cytoplasmic 1 gene (NFATc1, a regulator in osteoclast differentiation) [53,101,102]. However, the bioavailability of Ga remains a challenge because Ga salts are prone to form hydroxides and are potentially harmful upon consumption [100].

Magnesium (Mg), the second most abundant intracellular cation, stabilizes DNA and RNA structures and cell membranes and plays an essential role in maintaining the function of many enzymes as co-factors [74,186]. In skeletal bone, Mg deficiency contributes to impaired bone growth, disrupted mineral metabolism, decreased osteoblast, increased osteoclast cell number, and osteoporosis in young animals, with promoted inflammation [103,104,105,106,107]. Mg^2+^ is found to enhance the expression of the osteogenesis-related genes, production of extracellular matrix, and deposition of apatite crystal in undifferentiated MSCs and osteoblastic MSCs in vitro, possibly through the upregulation of hypoxia-inducible factor (HIF) and peroxisome proliferator-activated receptor-gamma coactivator-1 alpha (PGC-1α), respectively [108,109,110]. In vivo studies also showed enhanced bone regeneration with overexpressed osteogenic markers, OCN, Runx2, and IGF-I, around the implant in vivo [108,109,110]. Nevertheless, Mg^2+^ (up to 5 mM) competes with Ca^2+^ as an antagonist and forms an insoluble salt with pyrophosphate, causing mineralization defect and cell dysfunction [106,111,112].

Iron (Fe) is an essential element for humans. In skeletal bone, Fe contributes to the homeostasis of bone, with evidence showing that: Fe deficiency causes an overall loss in bone mass and density, with impaired biomechanical strength [113]; Fe overload is associated with disrupted differentiation and maturation of osteoblasts through the production of reactive oxygen species (ROS) [116]. Increased oxidative stress on cells elevates cytokine (tumor necrosis factor-alpha (TNF-α) and interleukin 6 (IL-6)) levels, leading to bone resorption and altered bone microarchitecture, and consequent bone loss and reduced biomechanical strength [114,115,116].

Manganese (Mn), an essential cofactor for many enzymes, is required in many biological processes. In skeletal bone, Mn deficiency causes abnormal bone growth (such as stunted bone growth and osteoporosis), while Mn overload leads to impaired bone development and neurotoxicity [21,117,118]. Incorporated in the inorganic biomaterial, Mn promotes the proliferation, adhesion, and spreading of osteoblasts, upregulates osteogenic-related gene expression (*ALP*, *BMP*), and accelerates collagen deposition [119,120,121,122]. Meanwhile, localized administration of Mn^2+^ exhibits an insulin-like effect, promoting angiogenesis and bone healing in vivo [123].

Selenium (Se) is an essential trace element in humans. The level of Se is correlated with bone metabolism and maintenance (Kashin-Beck disease), as well as well-being and protection against aging-related diseases [74,130,131]. Se deficiency leads to impaired bone and cartilage metabolism, osteopenia, and fracture susceptibility in several studies, both in vitro and in vivo, and even contributes to the progress of osteoporosis [124,125,126,127]. In contrast, Se overload is harmful to bone due to decreased mineral content, altered bone structure, and reduced biomechanical strength [128,129]. Both sides of the influence of Se on bone health are indications of the possible modulatory role of Se in the maintenance of skeleton bone. Se is likely involved in cellular responses in bone development by regulating microRNA in the formation of selenoprotein [187]. However, the roles of proteins and the influences of Se in bone metabolism at the cellular level remain unclear.

Silicon (Si), mainly found in the skeleton, is essential in bone metabolism [74,188,189]. In addition to the evidence of positive effects of dietary Si supplementation on bone health, promotive effects of Si-containing biomaterials in bone regeneration have also been extensively investigated [134,188,190,191,192]. Among all Si-containing biomaterials, bioactive glass is the most extensively studied. Bioglass 45S5 (BG), composed of SiO_2_, CaO, Na_2_O, and P_2_O_5_, was developed in the late 1960s [44]. Bioactive glasses are known for their excellence in bone bonding by forming an apatite layer on the surface and their capability to stimulate and promote the growth, proliferation, and differentiation of osteoblasts [132,133,134]. Soluble Si ions, in the form of orthosilicic acid, are found to stimulate osteogenic differentiation and enhance osteogenesis both in vitro and in vivo, possibly with the involvement of Wnt and Sonic Hedgehog (Shh) signaling pathways and the upregulation of miR-146a to antagonize the activation of NF-κB signaling pathway [135,136,137,138,139,140]. Si is also found to inhibit osteoclast phenotypic gene expression, osteoclast formation, and recruitment, as well as bone resorption in vitro, via reduced expression of receptor activator of nuclear factor-κB (RANK)/RANKL/osteoprotegerin (OPG) gene in osteoclast precursors or osteoclasts without the involvement of osteoblasts/stromal cells [51]. In addition, the inhibition effect of Si ions on osteoblast-induced osteoclastogenesis on murine macrophage cell line (RAW 264.7 cells) is also demonstrated in a co-culture system with human osteoblastic-like cell line (SaOS-2), resulting from increased secretion of OPG in osteoblastic-like cells and increased ratio of OPG/RANKL [138].

Strontium (Sr), mostly stored in skeleton bone, can exert many effects on bone metabolism at cellular and tissue levels in vitro and in vivo [145,193]. Strontium ranelate (SrRan), an organic salt of Sr, has been used as an anti-osteoporotic drug to treat osteoporosis for many years by shifting the balance between bone formation and resorption towards the former, although the mechanism remains unclear [142,146,194,195]. It is believed that SrRan enhances pre-osteoblast cell replication and collagen synthesis promotes osteoblast proliferation and differentiation and reduces bone resorption by reducing differentiation of osteoclasts and increasing osteoclast apoptosis, partly via the activation of CaSR due to the chemical similarity between Sr and Ca [141,142,145,146,147]. Sr^2+^ can activate the Wnt/β-catenin pathway or Ras/MAPK signaling pathway to upregulate the expression of osteogenic differentiation markers in cultured MSCs (such as ALP, Col-1, Runx2, OCN, and COX2), facilitate calcium deposition and nodule formation, and promote in vivo bone formation [48,143,144]. In osteoblasts, SrRan (1–5 mM) promotes cell survival and proliferation, depending on the activation of Akt- and ERK1/2- dependent signaling pathway via CaSR, or acts independently to modulate osteoblast viability and replication [78,141,142]. SrRan (0.1–1 mM) also induces differentiation in osteoblasts with observed overexpression of ALP, bone sialoprotein (BSP), OCN, and Runx2 [142,146]. Additionally, Sr^2+^ (20 and 100 μg/mL) was found to disturb mineralization in rodent MSCs [196]. In osteoblast-induced osteoclastogenesis, SrRan affects the balance between OPG and RANKL genes, further suppressing the NF-κB signaling in vitro and in vivo [148,149]. The direct impact of SrRan on osteoclasts involves the activation of NF-κB translocation and consequent mature cell apoptosis via the activation of the PKCβII signaling pathway in a dose-dependent manner; The inhibition of osteoclastic differentiation and resorptive activity is achieved by the reduction of carbonic anhydrase II (key enzyme for bone resorption) and vitronectin receptor (involved in the motility of osteoclast and maintenance of the sealing zone) expression [142,150,151].

Zinc [65] is an essential nutrient for the catalytic activity of over 200 enzymes in numerous biological processes, such as immune response, wound healing, and DNA and protein synthesis [197]. In skeletal bone, Zn is the most abundant trace metal and an essential cofactor for some bone metabolism-related enzymes, such as ALP (provides a phosphate source for bone mineralization) and collagenase and matrix metalloproteinases (essential in bone resorption and remodeling), indicating its role in maintaining bone mass, health and bone turnover rate [154,155]. In cultured hBMSCs, Zn^2+^ released from the implant material has been shown to promote cell viability, osteoblastic marker gene expression (Col-1, OCN, ALP, and BSP), matrix maturation, calcium deposition, and nodule formation [156]. Zn^2+^ is also believed to participate in bone metabolism as a signaling molecule modulating osteoblast and osteoclast cellular activities in vitro and in vivo [156,157,158]. In cultured osteoblasts, Zn^2+^ stimulates cell proliferation, differentiation, and mineralization by stimulating gene expression of various proteins associated with osteoblastic differentiation, such as type I collagen, ALP, OCN, OPN, and Runx2, and production of growth factors, such as IGF-1 or estrogen, related to enhanced cell proliferation [159,160]. In osteoclasts, Zn^2+^ acts as a potent inhibitor of resorptive activities [162]. The mechanisms of Zn^2+^ in promoting bone formation and suppressing bone resorption are achieved via the inhibition of the activation of TNFα driven NF-κB pathway [163].

In addition, the influence of some elements remains controversial due to conflicting results obtained in different studies, such as Fluorine (F), Lithium (Li), and Titanium (Ti). Some are being investigated due to observed positive effects in some therapeutical applications in bone diseases or biomaterial fabrication, such as Germanium (Ge), Niobium (Nb), and Vanadium (V) [74,185]. Some dose-related toxic metals in the body, released from inorganic biomaterials or dietary intake, have influenced bone resorption and formation through the modulations of bone cell activities. For example, Cobalt (Co) and Chromium (Cr), the major components of prosthetic implant materials for hip and knee joint replacements, are revealed to affect bone health with dissolved Co^2+^ and Cr^3+^ into the peri-implant bone and cause progressive local osteolysis [22,198,199]. Cytotoxicity of Co^2+^ and Cr^3+^ are well-established in osteoblast-like cells with altered morphology, decreased cell number, proliferation, and cellular activities with decreased release of OCN and collagen type I, reduced ALP activity and calcium deposition, possibly due to altered redox state [22,198,200,201]. Growth factors/cytokines, such as TGF-β1, TNF-α, IL-1β, and IL-6, secreted from osteoblasts under the stimulation of Co and Cr ions, lead to inflammation and further induce the maturation and differentiation of osteoclasts [199,200]. Nevertheless, the role of Co^2+^ in promoting vascularization in bone tissue is still worth pursuing because vascularization is also a critical component in bone regeneration [202,203,204,205].

## 5. Conclusions and Future Perspectives

A localized ionic environment is generated during bone remodeling, where the bone matrix containing organic molecules (growth factors, enzymes, etc.) and inorganic ions and ionic groups (Ca^2+^, PO_4_^3−^, Mg^2+^, etc.) is degraded. There is a wealth of evidence revealing the osteoinductive potentials of many individual ions, but few consider the effect of the ionic microenvironment. The composition and interactions among components in the localized ionic environment during bone remodeling seemed challenging to investigate. Still, we believe the mystery will be unveiled with more emerging state-of-the-art techniques and a deeper understanding of related fields. Additionally, more fundamental research is needed to address the effective species and dosage during biomaterial fabrication in the future. Nevertheless, the beneficial effects of ions in bone tissue engineering will shed light on the design of future inorganic biomaterials for bone regeneration. An inorganic biomaterial that provides a balanced combination of inorganic ions in a controlled and sustained way will potentially generate a desired ionic environment to regulate bone cell functions, resulting in optimal tissue regeneration.

## Figures and Tables

**Table 1:** Summary of major biological influences of bone homeostasis-related bioactive inorganics at physiological and cellular levels.

Ion	Related Disorders or Diseases	Effects on Cellular Activities		References
		+	−	
Ca	Deficiency: rickets, osteomalacia, and osteoporosis; Overload: poor bone health, kidney stone formation, and abnormal heart and brain function	MSC mineralization, osteoblast cell proliferation, survival and differentiation, osteoclast cell apoptosis	Osteoblast cell apoptosis, bone resorption	[9,74,76,77,78,79,80]
Pi	Deficiency: impaired bone mineralization, dysfunction in blood, muscle, central nervous system, cardio and respiratory system; Overload: kidney disease, cardiovascular disease, cancer, and skeletal disorder	Osteoblast and osteoclast cell apoptosis (high Pi level), osteoblastic differentiation and mineralization, bone resorption (low Pi level)	Bone resorption (at high Pi levels)	[50,75,81,82,83,84,85,86]
B	Deficiency: reduced osteogenesis, inhibited bone formation, decreased bone volume, and reduced mechanical strength	MSC and osteoblast osteogenic differentiation and mineralization	*	[49,87,88,89,90,91,92]
Cu	Deficiency: abnormal bone formation with impaired quality and strength, severe neurological issues, or liver diseases	angiogenesis, innate antibacterial property, extracellular matrix formation	*	[4,93,94,95,96,97,98,99]
Ga	*	Bone formation and mineralization	Osteoclast differentiation, bone resorption	[53,100,101,102]
Mg	Deficiency: impaired bone growth, disrupted mineral metabolism, and osteoporosis	MSC osteogenic differentiation and mineralization	Osteoblast differentiation (high Mg level)	[103,104,105,106,107,108,109,110,111,112]
Fe	Deficiency: overall loss in bone mass and density, impaired biomechanical strength Overload: metabolic bone diseases such as osteoporosis, altered bone microarchitecture, and reduced biomechanical strength	Bone resorption (high Fe level)	Osteoblast cell maturation and differentiation (high Fe level)	[113,114,115,116]
Mn	Deficiency: abnormal bone growth, such as stunted bone growth and osteoporosis; Overload: impaired bone development and neurotoxicity	Osteoblast proliferation, adhesion, and spreading, osteoblastic differentiation, collagen deposition, angiogenesis, and bone healing	*	[21,117,118,119,120,121,122,123]
Se	Deficiency: impaired bone and cartilage metabolism, osteopenia, osteoporosis, and Kashin-Beck disease (together with iodine); Overload: decreased mineral content, altered bone structure, and reduced biomechanical strength	**	*	[74,124,125,126,127,128,129,130,131]
Si	Deficiency: abnormal bone growth	Osteoblast cell growth, proliferation, and differentiation	Osteoclast formation, recruitment, and bone resorption, as well as osteoblast-induced osteoclastogenesis	[132,133,134,135,136,137,138,139,140]
Sr	*	Pre-osteoblast cell replication and collagen synthesis, osteoblast cell proliferation, survival, differentiation, mineralization, osteoclast cell apoptosis	Osteoclast cell survival, differentiation, osteoblast-induced osteoclastogenesis, and bone resorption	[48,78,141,142,143,144,145,146,147,148,149,150,151,152,153]
Zn	Deficiency: abnormal immune response, impaired wound healing, overall bone mass, and health, and bone turnover rate	MSC viability, osteoblastic differentiation, and mineralization, osteoblast cell proliferation, differentiation, and mineralization	Osteoclastogenesis and bone resorption	[154,155,156,157,158,159,160,161,162,163]

+ Promotive effect, − Inhibitive effect, * Not applicable, ** Unclear.

## Data Availability

Not applicable.
